# Ugonin J Acts as a SARS-CoV-2 3C-like Protease Inhibitor and Exhibits Anti-inflammatory Properties

**DOI:** 10.3389/fphar.2021.720018

**Published:** 2021-08-26

**Authors:** Wei-Chung Chiou, Hsu-Feng Lu, Nung-Yu Hsu, Tein-Yao Chang, Yuan-Fan Chin, Ping-Cheng Liu, Jir-Mehng Lo, Yeh B Wu, Jinn-Moon Yang, Cheng Huang

**Affiliations:** ^1^ Department of Biotechnology and Laboratory Science in Medicine, National Yang Ming Chiao Tung University, Taipei City, Taiwan; ^2^ Department of Medical Laboratory Science and Biotechnology, Asia University, Taichung City, Taiwan; ^3^ Department of Laboratory Medicine, China Medical University Hospital, Taichung City, Taiwan; ^4^ Institute of Bioinformatics and Systems Biology, National Yang Ming Chiao Tung University, Hsinchu City, Taiwan; ^5^ Institute of Preventive Medicine, National Defense Medical Center, New Taipei City, Taiwan; ^6^ Industrial Technology Research Institute, Biomedical Technology and Device Research Laboratories, Hsinchu City, Taiwan; ^7^ Arjil Biotech Holding Company Limited, Hsinchu City, Taiwan; ^8^ Department of Biological Science and Technology, College of Biological Science and Technology, National Yang Ming Chiao Tung University, Hsinchu City, Taiwan; ^9^ Center for Intelligent Drug Systems and Smart Bio-devices, National Yang Ming Chiao Tung University, Hsinchu City, Taiwan; ^10^ Faculty of Internal Medicine, College of Medicine, Kaohsiung Medical University, Kaohsiung City, Taiwan; ^11^ Hepatobiliary Division, Department of Internal Medicine, Kaohsiung Medical University Hospital, Kaohsiung Medical University, Kaohsiung City, Taiwan

**Keywords:** 3CL protease inhibitor, ugonin J, COVID-19, SARS-CoV-2, lung inflammation

## Abstract

Severe acute respiratory syndrome coronavirus 2 (SARS-CoV-2) infection causes severe “flu-like” symptoms that can progress to acute respiratory distress syndrome (ARDS), pneumonia, renal failure, and death. From the therapeutic perspective, 3-chymotrypsin-like protein (3CLpro) is a plausible target for direct-acting antiviral agents because of its indispensable role in viral replication. The flavonoid ugonin J (UJ) has been reported to have antioxidative and anti-inflammatory activities. However, the potential of UJ as an antiviral agent remains unexplored. In this study, we investigated the therapeutic activity of UJ against SARS-CoV-2 infection. Importantly, UJ has a distinct inhibitory activity against SARS-CoV-2 3CLpro, compared to luteolin, kaempferol, and isokaempferide. Specifically, UJ blocks the active site of SARS-CoV-2 3CLpro by forming hydrogen bonding and van der Waals interactions with H163, M165 and E166, G143 and C145, Q189, and P168 in subsites S1, S1′, S2, and S4, respectively. In addition, UJ forms strong, stable interactions with core pharmacophore anchors of SARS-CoV-2 3CLpro in a computational model. UJ shows consistent anti-inflammatory activity in inflamed human alveolar basal epithelial A549 cells. Furthermore, UJ has a 50% cytotoxic concentration (CC_50_) and a 50% effective concentration (EC_50)_ values of about 783 and 2.38 µM, respectively, with a selectivity index (SI) value of 329, in SARS-CoV-2-infected Vero E6 cells. Taken together, UJ is a direct-acting antiviral that obstructs the activity of a fundamental protease of SARS-CoV-2, offering the therapeutic potential for SARS-CoV-2 infection.

## Introduction

Severe acute respiratory syndrome coronavirus 2 (SARS-CoV-2) infection, giving rise to coronavirus disease 2019 (COVID-19), has threatened global public health and has had profound effects on the economy, psychology, and human behaviors (Chen et al., 2020). Infection with SARS-CoV-2 causes severe “flu-like” symptoms that can progress to acute respiratory distress syndrome (ARDS), pneumonia, renal failure, and death (Harrison et al., 2020). Common symptoms of COVID-19 include fever, dry cough, tiredness, and, in severe cases, dyspnea (Chen et al., 2020; Harrison et al., 2020). Current data from hospitalized patients indicate that older age, cardiovascular disease and metabolic disorders are risk factors for severe COVID-19, while healthy individuals remain equally susceptible to SARS-CoV-2 infection (Berlin et al., 2020; Harrison et al., 2020). Besides, severe lung pathology is associated with the inflammatory milieux and elevated production of cytokines and chemokines (Harrison et al., 2020). The dysregulated immune hyperactivation induced by SARS-CoV-2 infection is likely to advance to pneumonia, with lung damage, and acute systemic inflammatory symptoms (Fajgenbaum and June, 2020). Indeed, widespread inflammation in the lungs and excessive immune responses have also been observed in severe acute respiratory syndrome coronavirus (SARS-CoV) and Middle East respiratory syndrome coronavirus (MERS-CoV) infections (Fajgenbaum and June, 2020; Harrison et al., 2020; Quan et al., 2020).

Of great similarity to SARS-CoV, SARS-CoV-2 is an enveloped, positive-sense, single-stranded RNA (+ssRNA) Betacoronavirus (β CoV) that has a genome of about 30 kb, encoding nonstructural proteins (nsps), structural proteins, and several accessory proteins (Kim et al., 2020). From the therapeutic perspective, the nsps 3-chymotrypsin-like protein (3CLpro) and RNA-dependent RNA polymerase (RdRp), are considered to be plausible therapeutic targets of direct-acting antivirals, owing to their indispensable roles in viral replication (Li and De Clercq, 2020). In particular, the substrate-binding site of SARS-CoV-2 3CLpro is highly conserved across the β coronaviruses and alignment of the genomic sequences of SARS-CoV-2, SARS-CoV, and MERS-CoV reveals high-level conservation of the proteolytic sites (Zhang L. et al., 2020; Dai et al., 2020; Jin et al., 2020). Specifically, active SARS-CoV-2 3CLpro, a member of the cysteine protease family, constitutes two identical monomers, each containing three structural domains, where the first two domains (domain I: 8–101 and II: 102–184) form a chymotrypsin fold, and the third domain (domain III: 201–303) forms a globular α-helical structure (Chan et al., 2020; Jin et al., 2020). Importantly, H41 and C145 are the catalytic dyad of SARS-CoV-2 3CLpro (Jin et al., 2020), and the formation of the S1 subsite of the substrate-binding site requires association between the N-terminal residue (N-finger) of one and the E166 residue of the other (Zhang L. et al., 2020). On the other hand, the most variable regions of 3CLpro in known CoVs reside in domain III and the surface loops (Jin et al., 2020).

With the chemical diversity of phytochemicals, these natural compounds are of great interest in antiviral research and may have therapeutic potential against coronaviral infections (Bhuiyan et al., 2020; Mani et al., 2020). Polyphenols are identified as efficient small molecules against coronavirus inhibitors (Mani et al., 2020). Indeed, flavonoids, including luteolin, quercetin, epigallocatechin gallate, and kaempferol, have been reported to inhibit the proteolytic activity of SARS-CoV 3CLpro (Jo et al., 2020b; Chiou et al., 2021). Meanwhile, quercetin, baicalin, epigallocatechin gallate, and pentagalloylglucose were identified as SARS-CoV-2 3CLpro inhibitors *in vitro* (Abian et al., 2020; Jo et al., 2020a; Chiou et al., 2021). In addition, luteolin and kaempferol are hit compounds in computational screening, potentially binding to the substrate-binding site of SARS-CoV-2 3CLpro (Cherrak et al., 2020; Yu et al., 2020). Flavonoid ugonin J (UJ) has been reported to possess antioxidative and anti-inflammatory activities (Huang et al., 2003; Huang et al., 2009; Huang et al., 2017). However, the antiviral activity of UJ against SARS-CoV-2 remains to be elucidated.

In this study, we investigated the inhibitory activity of UJ against SARS-CoV-2 3CLpro *in vitro*, along with luteolin (Lu), kaempferol (Kae), and isokaempferide (Ikae). To determine the protease activity, the fluorescence signal emitted from cleaved intramolecularly quenched fluorescent (IQF) peptide substrate was recorded. Coupling this with dose-response curves, we unraveled the inhibitory mechanism of UJ in the substrate-binding site of SARS-CoV-2 3CLpro, using GEMDOCK molecular modeling software (Hsu et al., 2011; Pathak et al., 2021). UJ was further evaluated in inflamed human alveolar basal epithelial A549 cells and SARS-CoV-2-infected Vero E6 cells.

## Materials and Methods

### Chemicals

Ugonin J, kindly provided by Dr. Yu-Ling Huang (National Research Institute of Chinese Medicine, Taiwan), was prepared following the protocol described previously (Huang et al., 2003; Huang et al., 2009) and had a purity of >98%. Luteolin (Lu; L9283), kaempferol (Kae; 60010), isokaempferide (Ikae; SMB00179), and dexamethasone (Dex; D4902) were purchased from Sigma-Aldrich, United States. Remdesivir (Rem; GS-5734) (S8932) and boceprevir (S3733) were purchased from Selleckchem, United States.

### Protein Purification of SARS-CoV-2 3-Chymotrypsin-Like Protein

Recombinant proteins SARS-CoV 3CLpro and SARS-CoV-2 3CLpro were expressed in and purified from the *E. coli* as described previously (Kuang et al., 2005; Chiou et al., 2021).

### Protease Activity Assay and Dose-Response Curve Analysis

Following the protocol published previously (Chiou et al., 2021), 0.125 µM 3CLpro of SARS-CoV-2 was incubated with a compound at the indicated concentration at 37°C for one hour. Later, IQF peptide substrate was added to a final concentration of 1.25 µM, followed by 3-h incubation at 37°C. RFU measurements were made to obtain the relative protease activity. Values of relative protease activity in the presence or absence of a compound were fitted to a normalized dose-response (variable slope) model in GraphPad Prism 7.03 (GraphPad, United States) for IC_50_ characterization.

### Molecular Modeling in the Pharmacophore Anchor Model

The 3D structures of UJ, Lu, Kae and Ikae were downloaded as SDF files from PubChem (Kim et al., 2019) and converted to MOL files in OpenBabel software (O’Boyle et al., 2011). Then, molecular docking of compounds UJ, Lu, Kae and Ikae to SARS-CoV-2 3CLpro [Protein Data Bank: 6LU7 (Jin et al., 2020)] was performed using GEMDOCK molecular modeling software, according to our previous study (Hsu et al., 2011). To build the pharmacophore anchor model in SiMMap (Pathak et al., 2021), the top 3,000 docking energy poses were introduced to identify consensus interactions by profile in matrix M (T), with type electrostatic bond (E), hydrogen bond (H), and van der Waals forces (V) of size i (number of compounds) × j (number of interaction residues). Each anchor has one or more of the E-H-V type of interactions. In SiMMap, the standard deviation and the mean were calculated from 1,000 times random shuffling, with a Z-score of 1.645 or greater, to identify physical-chemical properties for interactions and moieties.

### Cell Lines, SARS-CoV-2 Strain, and Cell Viability

A549 cells (CCL-185™) and Vero E6 cells (CRL-158™) were purchased from ATCC®. Cells were maintained in Dulbecco’s modified Eagle’s medium (Gibco, United States) with 10% FBS (Gibco, United States) at 37°C in an atmosphere of >95% humidity and 5% CO_2_ and were passaged every 2–3 days. The SARS-CoV-2 strain 3586 (TSGH_15 GISAID accession number EPI_ISL_436100) was isolated from the Institute of Preventive Medicine, National Defense Medical Center and was amplified in Vero E6 cells. The viral titer was determined by plaque assays. Experiments involving live SARS-CoV-2 virus were carried out in a BSL-3 laboratory. To determine the cytotoxicity of compounds of interest, Vero E6 cells were seeded at 5 × 10^3^ in 96-well plates (SPL, Korea) and the cell viability assay was performed as described previously (Chiang et al., 2020).

### Real-Time Polymerase Chain Reaction and Western Blot Analysis

Real-time polymerase chain reaction (RT-PCR) was performed using a published protocol (Chiang et al., 2020). Briefly, RNA isolation was performed using the TRIzol reagent manual (Ambion, United States). Nucleic acid quantification was measured by a NanoDrop ND-1000 Spectrophotometer (Thermo Fisher Scientific, United States). Equal RNA samples were reverse-transcribed with the RevertAid First Strand cDNA Synthesis kit (Thermo Fisher Scientific, United States). Quantitative PCR was performed using SYBR Green PCR Master Mix from Applied Biosystems, United States, and the Ct value of genes of interest was measured by Applied Biosystems™ StepOne™ Real-Time PCR System (United States) without autocorrection. The primer pairs used in this study are listed in Supplementary Table S1. Relative mRNA expression levels were calculated using the ΔΔCt method.

For western blot analysis, an equal amount of the samples were resolved by SDS-PAGE, followed by transfer to PVDF membranes, following the protocol published previously (Chiang et al., 2020). Antibodies against TNF-α (GTX110520) and β-actin (GTX109639) were purchased from GeneTex, Taiwan. HRP-conjugated secondary antibodies were from Jackson ImmunoResearch Laboratories, Inc., United States. WesternBright® ECL kits were used for protein visualization (Advansta Inc., United States). Levels of protein expression were quantified in Fiji (Schindelin et al., 2012).

### Plaque Assay

For plaque assay, Vero E6 cells were seeded at 4 × 10^5^/well in 12-well cell culture plates (Greiner, Germany) the day before virus infection. Cells were pretreated with the test compound for an hour at 37°C before virus infection. SARS-CoV-2 was added at 100 PFU to the cell monolayer for an hour at 37°C in the presence of the test compound. Later, viruses were removed, followed by washing with 1× PBS. The cell monolayer was then covered with culture medium containing 1% methylcellulose (M0387, Sigma-Aldrich, United States) and the test compound for 3 days at 37°C. The cells were then fixed with 10% formaldehyde (Sigma-Aldrich, United States) overnight and stained with crystal violet (C6158, Sigma-Aldrich, United States). Percent inhibition was calculated as [1—(CT (compound treatment)/VC (virus control))] × 100%, where CT and VC refer to the plaque counts in the presence and absence of treatment with the compound.

### Statistical Analysis

Data (*N* = 3) were analyzed and plotted with GraphPad Prism 7.03 (GraphPad, United States). Values were expressed as the mean ± standard error mean (SEM). Student’s t-tests were performed to determine the statistical significance between the two groups. One-way ANOVA post hoc Dunnett’s multiple comparison tests were used to determine the statistical significance between the control group and two or more treated groups. Statistical significance was denoted by asterisks or hashtags (e.g., *, *p* < 0.05; **, *p* < 0.01; ***, *p* < 0.001).

## Results

### Ugonin J Inhibited the Protease Activity of SARS-CoV-2 3-Chymotrypsin-Like Protein, With a One-Digit Micromolar IC_50_ Value Against SARS-CoV-2 3-Chymotrypsin-Like Protein

**FIGURE 1:**
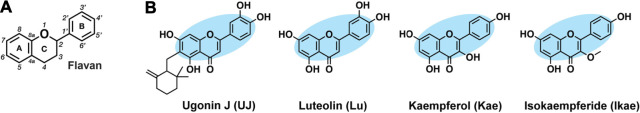
Chemical structures of UJ, Lu, Kae, and Ikae. **(A)** Flavans contain two aromatic rings (A, B) and an oxygen-containing heterocyclic ring (C). **(B)** Ugonin J (UJ), luteolin (Lu), kaempferol (kae), and isokaempferide (Ikae). Blue shading indicates the core structure. Hydrogens attached to carbons are omitted.

Flavan is the basic structure of flavonoids, comprising two aromatic rings (ring A and B) and a heterocyclic ring (ring C), as depicted in Figure 1A. The chemical structures of UJ, Lu, Kae, and Ikae are shown in Figure 1B. Luteolin has four hydroxyl groups at the C5, C7, C3′, and C4′ positions and a carbonyl group at the C4 position. Closely resembling luteolin, kaempferol and isokaempferide lack a hydroxyl group at the C3’ position but have an additional hydroxyl group and methoxy group, respectively, at the C3 position. UJ has an ethyl-(2,2-dimethyl-6-methylenecyclohexyl) group at the C6 position.

**FIGURE 2:**
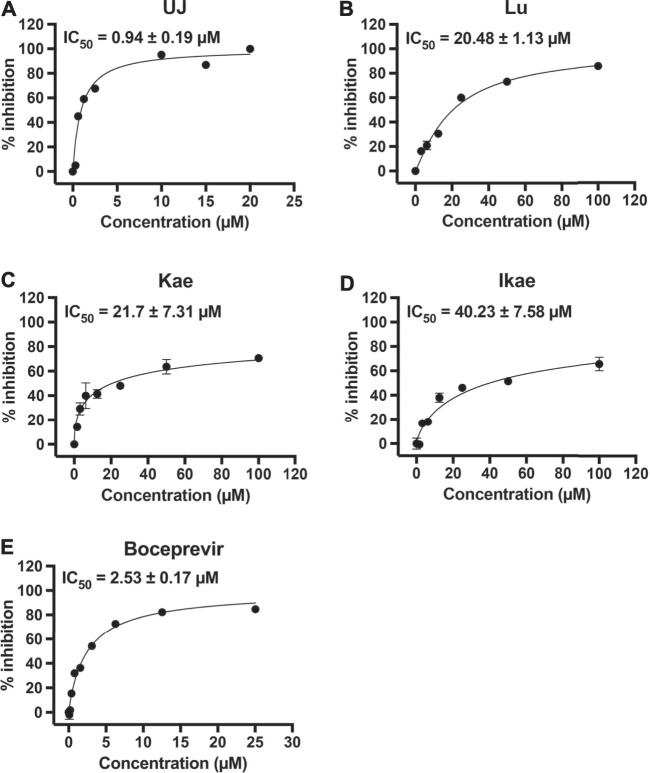
The effect of UJ, Lu, Kae, and Ikae on SARS-CoV-2 3CLpro activity. **(A–E)** SARS-CoV-2 3CLpro was incubated with or without UJ, Lu, Kae, Ikae, and boceprevir (0–100 µM) and the protease activity was measured. Dose-response curves of UJ **(A)**, Lu **(B)**, Kae **(C)**, Ikae **(D)**, and boceprevir **(E)** against SARS-CoV-2 3CLpro are shown. Graphs show the percent inhibition at each concentration. Data (*N* = 3) are shown as the mean ± SEM.

To characterize the half-maximal concentration IC_50_ values of UJ, Lu, Kae, and Ikae against SARS-CoV-2 3CLpro, the relative activities at multiple compound concentrations were determined for dose-response curves. Previous studies demonstrated that boceprevir inhibits the proteolytic activity of SARS-CoV-2 3CLpro (Fu et al., 2020; Ma et al., 2020; Pathak et al., 2021). UJ had the lowest IC_50_ value, 0.94 ± 0.19 µM (Figure 2A), while the IC_50_ values of Lu, Kae and Ikae were greater than 20 µM (Figures 2B–D). As a reference molecule, boceprevir had an IC50 value of 2.53 ± 0.17 µM against SARS-CoV-2 3CLpro (Figure 2E). The inhibitory activity of 12.5 µM UJ, Lu, Kae, and Ikae against SARS-CoV and SARS-CoV-2 3CLpro was evaluated in parallel (data not shown). UJ at 12.5 µM potently inhibited the proteolytic activity of both 3CLpro, down from 100% to about 15%. Meanwhile, Lu, Kae, and Ikae at 12.5 µM reduced relative SARS-CoV and SARS-CoV-2 3CLpro activity from 100% to 60–70%. Taken together, UJ significantly suppressed the protease activity of both SARS-CoV and SARS-CoV-2 3CLpro.

### Ugonin J Forms Multiple Interactions With the Core Pharmacophore Anchors in the SARS-CoV-2 3-Chymotrypsin-Like Protein Active Site

**FIGURE 3:**
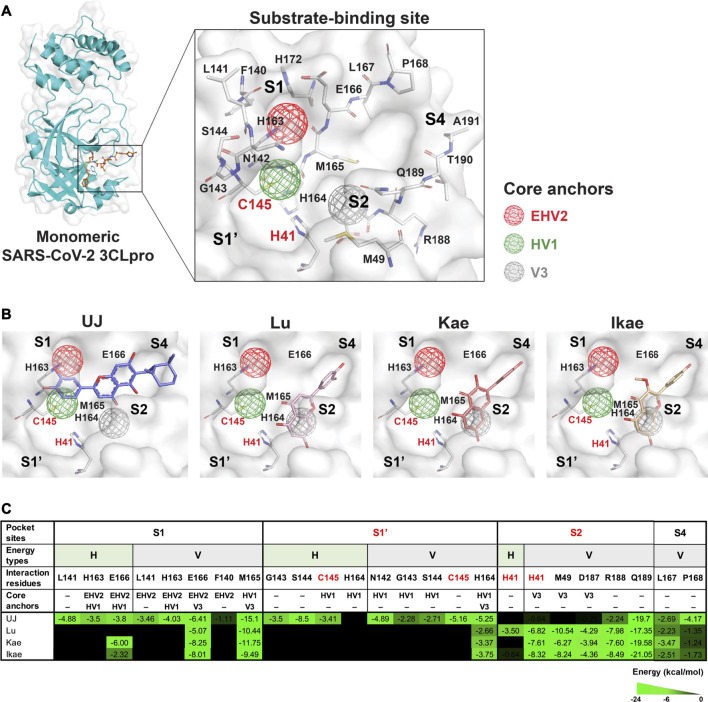
Molecular and pharmacophore modeling of UJ in the substrate-binding site of SARS-CoV-2 3CLpro. **(A)** A protomer of SARS-CoV-2 3CLpro and its substrate-binding site. Amino acids involved in substrate binding are labeled. Hollow spheres indicate the area of the core pharmacophore anchors EHV2 (red), HV1 (green), and V3 (gray). **(B)** Molecular docking to SARS-CoV-2 3CLpro. Key amino acid residues included in the core pharmacophore anchors are labeled. **(C)** Interaction energy (kcal/mol) between the compound and the amino acids involved in substrate binding. Subsites containing the catalytic residues (S1′ and S2) are in red. Energy types, hydrogen bonding (H) and/or van der Waals forces (V), are indicated above each interaction energy. Residues located in the core pharmacophore anchors are indicated. The strength of the interaction is shaded in green.

The substrate-binding site of SARS-CoV-2 3CLpro includes four subsites, S1, S1′, S2, and S4, and the amino acids involved in substrate binding have been elucidated (Jin et al., 2020). Residues H41 and C145 are the catalytic dyad of SARS-CoV-2 3CLpro (Figure 3A). Importantly, the core pharmacophore anchors EHV2, HV1, and V3 in the active site of SARS-CoV-2 3CLpro have been reported and the residue E166 plays a crucial role in SARS-CoV-2 3CLpro dimerization (Zhang L. et al., 2020; Pathak et al., 2021). SARS-CoV-2 3CLpro inhibitors block proteolysis through consistent interactions with the residues in pharmacophore anchors. To elucidate SARS-CoV-2 3CLpro inhibition, the docking positions of UJ, Lu, Kae, and Ikae, simulated using GEMDOCK molecular modeling software, were analyzed for interactions with the core pharmacophore anchors in SiMMap. From the molecular docking results shown in Figure 3B, UJ formed stable hydrogen bonding with the residue C145 in the HV1 anchor and strong van der Waals forces with residue M165 in the HV1 and V3 anchors and a stable interaction with residue E166 in the EHV2 and V3 anchors and residue H164 in the HV1 and V3 anchors. On the other hand, Lu, Kae, and Ikae interacted with the catalytic residue H41 in the V3 anchor by van der Waals forces, with stable van der Waals interactions with E166 in the HV1 and EHV2 anchors and M165 in the HV1 and V3 anchors. The interaction energy (kcal/mol) between the compound and the amino acids involved in substrate-binding is shown in Figure 3C, along with the core pharmacophore anchors identified. UJ interacted with the catalytic residue C145 in the HV1 anchor and formed multiple types of interactions with the core pharmacophore anchors in subsites S1 and S1’. As for Lu, Kae, and Ikae, these compounds mainly interacted with the residues in subsites S2 and S4 *via* van der Waals forces. To validate the molecular docking methodology, linear regression analysis was performed to characterize the relationship between the experimental data (e.g., IC_50_ values) and the binding energy from molecular modeling (Isaacs et al., 2020). The linear relationship between the IC_50_ values and the binding energy from GEMDOCK and molecular dynamics (MD) simulation have an R-square value of 0.8843 and 0.9096, respectively, as shown in Supplementary Figure S1. Taken together, compared to Lu, Kae, or Ikae, UJ had relatively more interactions with the residues in the core pharmacophore anchors in SARS-CoV-2 3CLpro.

### Ugonin J Attenuated Lipopolysaccharide-Stimulated Inflammation in A549 Cells

**FIGURE 4:**
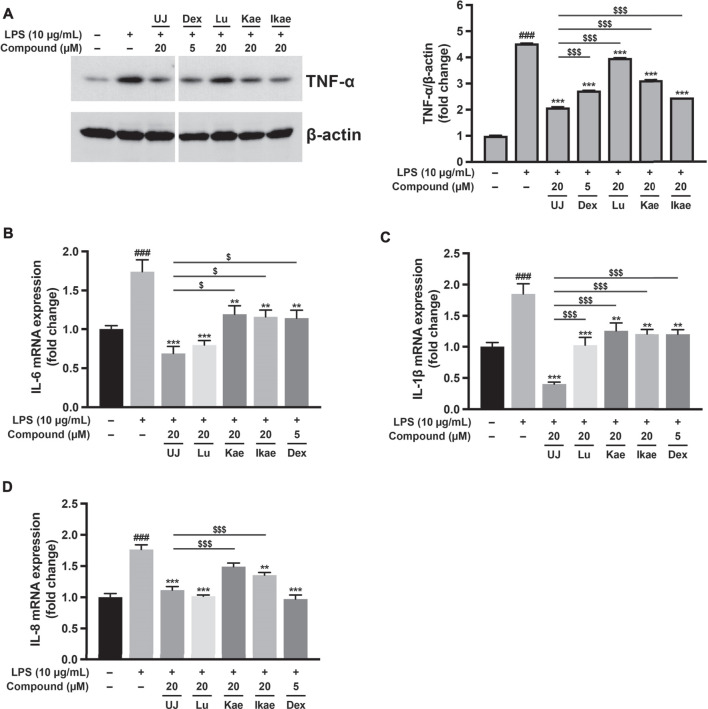
The anti-inflammatory effects of ugonins on LPS-stimulated inflammation of A549 cells. A549 cells were treated with UJ, Lu, Kae, or Ikae at 20 µM one hour before 10 μg/ml LPS stimulation and incubated for a further 24 h. Cells were harvested for western blot analysis and RT-PCR. Dex at 5 µM was used as a reference for anti-inflammatory activity. All groups were adjusted with the drug vehicle accordingly. Protein expression levels of TNF-α shown in **(A)** are normalized by the protein loading control (β-actin). mRNA expression levels of IL-6 **(B)**, IL-1β **(C)**, and IL-8 **(D)** are shown. Data (*N* = 3) are shown as the mean ± SEM. Student’s t-tests were performed to determine the statistical significance between two groups: the mock group vs. the LPS group (#, *p* < 0.05; ##, *p* < 0.01; ###, *p* < 0.001) and the UJ group vs. the other drug group ($, *p* < 0.05; $$, *p* < 0.01; $$$, *p* < 0.001). One-way ANOVA with Dunnett’s multiple comparisons tests were performed to determine the statistical significance between the LPS group and the drug-treated groups (*, *p* < 0.05; **, *p* < 0.01; ***, *p* < 0.001).

Human alveolar basal epithelial A549 cells have been used to investigate several lung diseases at the cellular level, including endotoxin-induced acute pneumonia (Zhang J. et al., 2020). To evaluate the anti-inflammatory effect of UJ, A549 cells were induced with lipopolysaccharide (LPS) for 24 h with or without treatment with UJ. Dexamethasone (Dex), an anti-inflammatory agent (Chen et al., 2021), was included as a positive control. As shown in Figure 4A, LPS stimulation upregulated the protein levels of tumor necrosis factor alpha (TNF-α) by more than 4-fold, compared with the control group. UJ treatment at 20 µM significantly reduced the TNF-α protein expression level by more than 2-fold in LPS-stimulated A549 cells. In addition, the expression levels of proinflammatory genes, including interleukin 1β (IL-1β), IL-6 and IL-8 increased by 1.5- to 2-fold in LPS-stimulated A549 cells, compared with the control group (Figures 4B–D). UJ treatment significantly downregulated the mRNA expression levels of IL-6, IL-1 β, and IL-8 by more than 1-fold at 20 µM in LPS-stimulated A549 cells. Meanwhile, Lu, Kae, and Ikae at 20 µM had a relatively moderate potency with respect to the reduction of the levels of these four proinflammatory factors, compared to UJ treatment, indicating that UJ had a potent activity, mitigating LPS-stimulated inflammation in A549 cells. Compared to the positive control (Dex at 5 µM), UJ treatment at 20 µM gave rise to a greater decrease in the TNF-α protein level and IL-6 and IL-8 mRNA. Taken together, UJ exhibited a relatively consistent anti-inflammatory activity in LPS-stimulated A549 cells.

### Ugonin J Showed Anti-SARS-CoV-2 Activities in Vero E6 Cells

**FIGURE 5:**
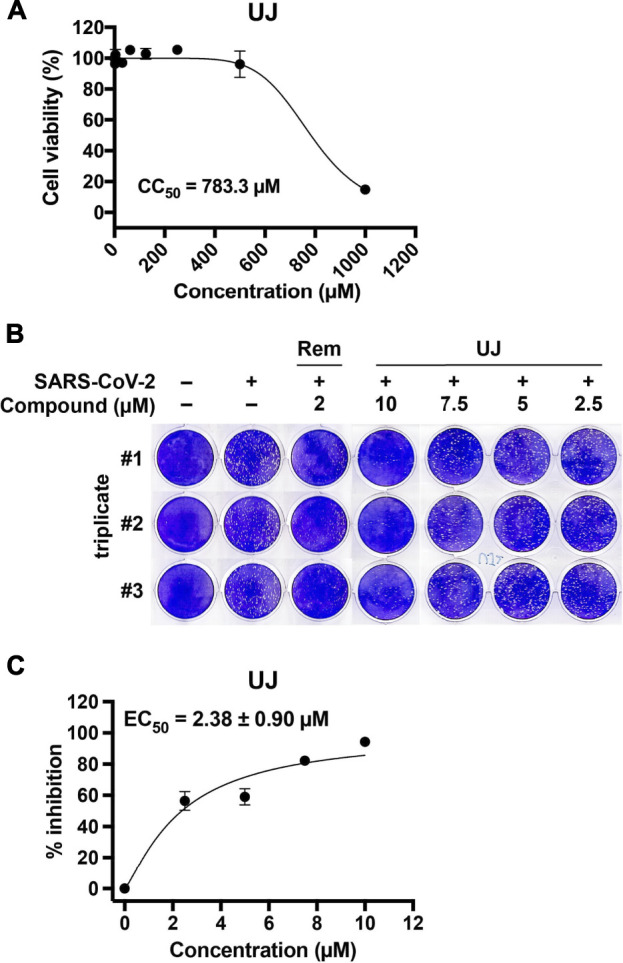
The cytotoxic and anti-SARS-CoV-2 activities of UJ in Vero E6 cells. **(A)** Effect of UJ on the viability of Vero E6 cells. Cells were incubated in culture medium containing 0–1,000 µM UJ for 48 h. The half-maximal cytotoxic concentration (CC_50_) of UJ is shown. **(B)** Plaque assay in SARS-CoV-2-infected Vero E6 cells. **(C)** The half-maximal effective concentration (EC_50_) of UJ against SARS-CoV-2 infection in Vero E6 cells. Data (*N* = 3) are shown as the mean ± SEM.

To investigate further the effect of UJ on SARS-CoV-2 infection, the half-maximal cytotoxic concentration (CC_50_) of UJ in Vero E6 cells was determined first. As shown in Figure 5A, UJ has a CC_50_ value of about 783.3 µM in Vero E6 cells. In Figure 5B, UJ reduced the plaque size and number dose-dependently. In particular, pretreatment with UJ at 10 µM manifested a near complete prevention of SARS-CoV-2 infection of Vero E6 cells. In parallel, the reference drug Rem was applied at 2 µM and resulted in about 95% inhibition of SARS-CoV-2 infection of Vero E6 cells. As shown in Figure 5C, the half-maximal effective concentration (EC_50_) value of UJ against SARS-CoV-2 infection of Vero E6 cells was 2.38 ± 0.90 µM and the selectivity index (SI) of UJ, CC_50_/EC_50_, was about 329. Taken together, UJ shows efficient anti-SARS-CoV-2 activity in SARS-CoV-2-infected Vero E6 cells.

## Discussion

The rapidly evolving developments of the COVID-19 pandemic highlight the risks of zoonotic virus spillovers and the fundamental need for proactive approaches to shorten the gap between outbreak and response (Harrison et al., 2020). Therapeutic options for SARS-CoV-2 infection include direct-acting antivirals, such as small molecules, neutralizing antibodies and RNA-based therapeutics, and host-factor therapeutics (Meganck and Baric, 2021). Importantly, direct-acting antivirals may select drug-resistance mutations and host-factor therapeutics may have greater toxicity, because of the lack of selectivity for infected cells (Artese et al., 2020; Meganck and Baric, 2021). Based on the therapeutic experience with human immunodeficiency virus (HIV) and hepatitis C virus (HCV), it is reasonable to believe that drug combinations and cocktails can offer substantial clinical benefits in the context of the COVID-19 pandemic (Weisberg et al., 2020). Indeed, drug combinations have been extensively explored in fighting SARS-CoV-2 infection. Baricitinib in combination with remdesivir, for example, has been approved by the US Food and Drug Administration (FDA), under Emergency Use Authorization (EUA), as a COVID-19 treatment for hospitalized adults, especially those receiving high-flow oxygen or noninvasive ventilation (Kalil et al., 2021; Meganck and Baric, 2021). On the other hand, triple combination treatment, consisting of interferon beta-1b, lopinavir-ritonavir, and ribavirin, alleviated symptoms and shortening the duration of viral shedding and hospital stay in mildly-to-moderately ill COVID-19 patients (Hung et al., 2020). An antibody cocktail consisting of casirivimab and imdevimab has been approved by the US FDA, under EUA, as a treatment for mildly-to-moderately ill COVID-19 patients (Wang et al., 2021). Given that these COVID-19 therapeutic combinations, approved by the US FDA under EUA, do not include direct-acting antivirals targeting SARS-CoV-2 3CLpro; it is of great interest to identify small-molecule agents that have potent inhibitory activity against coronaviral infections. For instance, boceprevir, an HCV NS3–4A protease inhibitor, shows great potential for treating SARS-CoV-2 infections (Fu et al., 2020; Ma et al., 2020; Pathak et al., 2021). Nonetheless, the chemical diversity of phytochemicals provides a plethora of chemical backbones and functional groups that may be exploited in devising novel, broad-spectrum protease inhibitors.

In this study, we investigated whether UJ shows promise for SARS-CoV-2 infection. UJ, a major constituent of HZ, showed excellent activity against the proteolytic activity of both SARS-CoV and SARS-CoV-2 3CLpro. Specifically, the IC_50_ value of UJ against SARS-CoV-2 3CLpro was about 0.94 µM. In molecular modeling, UJ interacted with several core and consensus anchor residues in the active site of SARS-CoV-2 3CLpro (Shitrit et al., 2020; Pathak et al., 2021) (i.e., H163, M165 and E166, G143 and C145, Q189, and P168 in subsites S1, S1′, S2, and S4, respectively) *via* hydrogen bonding and/or van der Waals interactions, thereby blocking the peptide substrate from accessing the active site. In contrast, the core and consensus residues that Lu, Kae, and Ikae interacted with were M165 and E166, H41, M49 and Q189, and P168 in subsites S1, S2, and S4, respectively, *via* primarily van der Waals forces. In particular, forming hydrogen bonding with H163 and E166 has been regarded as an important feature for potent inhibition of the viral polypeptide cleavage process of SARS-CoV-2 3CLpro (Shitrit et al., 2020).

Besides, UJ interactions on the surface of SARS-CoV-2 3CLpro were investigated in MD simulation. Molecular mechanics energies combined with the Poisson−Boltzmann (MM-PBSA) or generalized Born (MM-GBSA) (Miller et al., 2012) and CHARMM (Brooks et al., 2009) are widely applied in the field of MD to calculate free energies of molecules, improving the reliability of computational models in drug discovery. The former is an efficient, reliable approach to identify potential molecules in solution (Sarukhanyan et al., 2018), and the latter has also been extended to simulate drug-target interactions in medicinally relevant systems (Vanommeslaeghe et al., 2010). Here, MD simulation was performed in BIOVIA Discovery Studio 2018 using CHARMM. As shown in Supplementary Figure S2A, the root-mean-square deviation (RMSD) of SARS-CoV-2 3CLpro main chain residue with UJ was below 2 Å in the 10 ns MD simulation, indicating that the binding conformation of UJ to SARS-CoV-2 3CLpro is stable. Meanwhile, the corresponding conformation energy in this MD simulation was shown in Supplementary Figure S2B. However, an MD simulation for more than 50 ns needs further investigation to clarify the binding affinity of UJ for SARS-CoV-2 3CLpro.

Regarding the pragmatic use of UJ, we investigated its anti-inflammatory activity in LPS-stimulated A549 cells and their anti-SARS-CoV-2 activity in Vero E6 cells. Corresponding to previous studies (Huang et al., 2003; Huang et al., 2009; Huang et al., 2017; Baier et al., 2018), UJ, Lu, Kae, and Ikae showed anti-inflammatory activities in LPS-stimulated A549 cells. In particular, UJ demonstrated a consistent anti-inflammatory effect with respect to the expression levels of TNFα, IL-6, IL-1β, and IL-8 in LPS-stimulated A549 cells. Meanwhile, UJ effectively prevented SARS-CoV-2 infection of Vero E6 cells, with an EC_50_ value of about 2.38 µM and an SI value of about 329. Concerning the incidence of dysregulated immune hyperactivation (Fajgenbaum and June, 2020), the dual bioactivity of UJ, anti-inflammatory and anti-SARS-CoV-2 activities, highlights the potential of UJ for fighting SARS-CoV-2 infection.

In conclusion, we show that UJ not only inhibits SARS-CoV-2 3CLpro activity but also prevents SARS-CoV-2 infection. Specifically, UJ interacts with multiple core and consensus anchor residues involved in the core pharmacophore anchors of SARS-CoV-2 3CLpro, *via* hydrogen bonding and/or van der Waals interactions. Acting as a direct-acting antiviral, UJ obstructs the activity of a fundamental protease of SARS-CoV-2. In SARS-CoV-2-infected Vero E6 cells, UJ had an SI value of 329, a CC_50_ of 783 μM, and an EC_50_ of 2.38 µM. Altogether, UJ is a novel viral protease inhibitor that presents the therapeutic potential for SARS-CoV-2 infection.

## Data Availability

The original contributions presented in the study are included in the article/Supplementary Material, further inquiries can be directed to the corresponding author.
