# Supplementary material for: Ugonin J Acts as a SARS-CoV-2 3C-like Protease Inhibitor and Exhibits Anti-inflammatory Properties
Source: Front Pharmacol. 2021 Aug 26;12:720018. doi: 10.3389/fphar.2021.720018 (PMC8427442; doi:10.3389/fphar.2021.720018)
Supplement: Supplementary file 1 [file DataSheet1.pdf]

Supplementary Material

1 Supplementary Tables

Table S1. Primers

Name	Forward sequence (5'- to 3'-end)	Reverse sequence (5'- to 3'-end)
h-TNF-α	AGCCCATGTTGTAGCAAACC	TGAGGTACAGGCCCTCTGAT
h-IL-1β	AAACAGATGAAGTGCTCCTTCCAGG	TGGAGAACACCACTTGTTGCTCCA
h-IL-6	GGCTGAAAAAGATGGATGCT	CCTGCTTCACCACCTTCTG
h-IL-8	TGGCTCTCTTGGCAGCCTTC	TGCACCCAGTTTTCCTTGGG
h-GAPDH	GAAGGTGAAGGTCGGAGT	GAAGATGGTGATGGGATTTC

2 Supplementary Figures

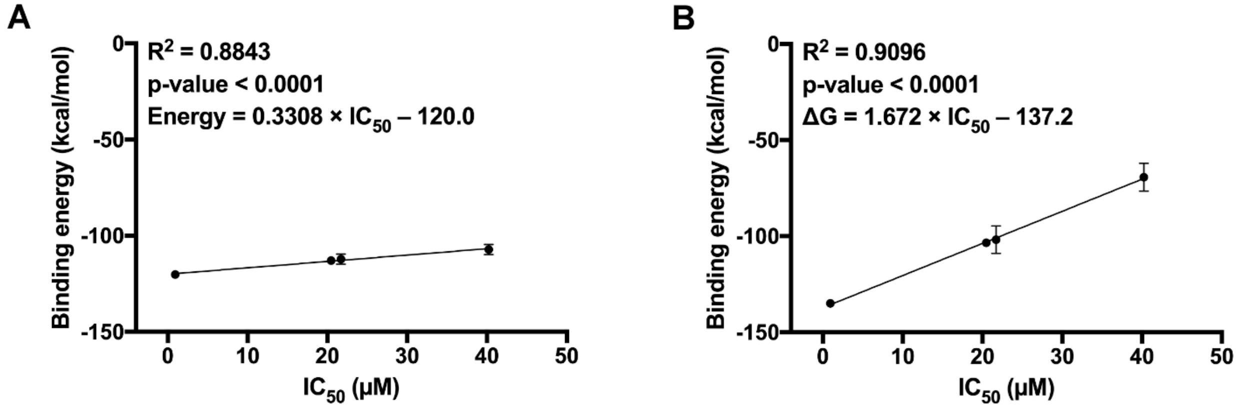

Figure S1 | The linear relationship between the binding energy and IC_{50} values. Correlation between the IC_{50} values and binding energy from **(A)** GEMDOCK molecular modeling software and **(B)** molecular dynamics simulation. Points from left to right represent UJ, luteolin, kaempferol, and isokaempferide. The linear regression analysis was performed using simple linear regression in GraphPad Prism 7.03. Molecular dynamics (MD) was simulated in BIOVIA Discovery Studio 2018 using CHARMM to consider Gibbs free energy of binding of these four compounds in a duration of 1 ns. With the parameters of Minimization, Minimization2, and Target Temperature set to Steepest Descent with max steps of 1000, Conjugate Gradient with max steps of 2000, and 300 K, respectively, the binding energy of each conformation was generated every 2 ps.

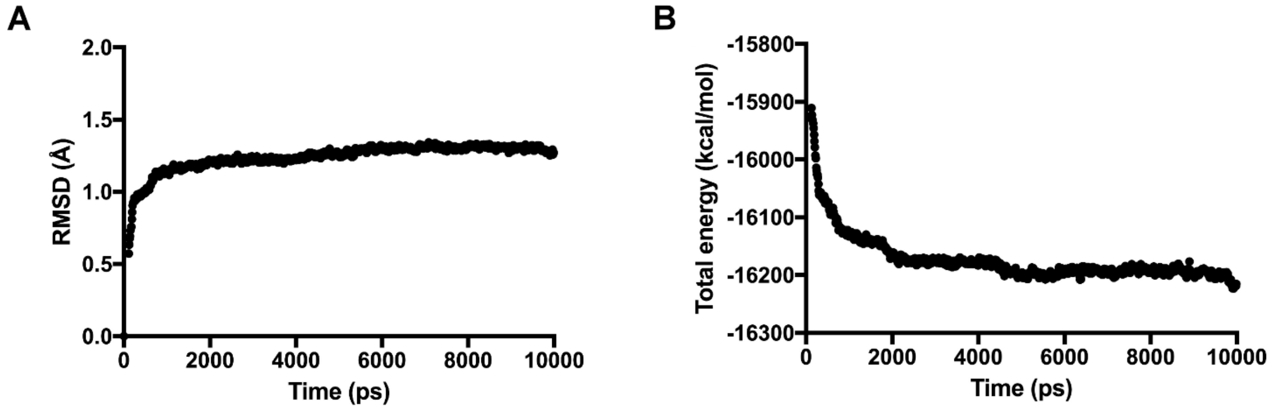

Figure S2 | RMSD and conformation energy versus time plot during the 10 ns MD simulation. **(A)** Root-square-mean deviation (RMSD). Following the settings described in Supplementary Figure 1, CHARMM was employed to perform the 10 ns MD simulation, in which each conformation of UJ to SARS-CoV-2 3CLpro was saved every 10 ps. Compared with the original docking pose, the RMSD was determined in a unit of 10^{-10} (Å). **(B)** Conformation energy. The corresponding conformation energy (kcal/mol) of UJ to SARS-CoV-2 3CLpro during the 10 ns MD simulation was recorded.
